# Research on characteristic properties of ASiGe nanoribbons materials for nanoelectronics and optoelectronics applications

**DOI:** 10.1098/rsos.241793

**Published:** 2025-02-19

**Authors:** Tran Minh Tien

**Affiliations:** ^1^ Thu Dau Mot University, Binh Duong province, Thu Dau Mot, Vietnam

**Keywords:** VASP, armchair SiGe nanoribbons, electronic properties, optical properties

## Abstract

This article presents the properties of armchair SiGe nanoribbon (ASiGeNR) configurations with varying ribbon widths. Using the Vienna Ab initio Simulation Package calculation program, several basic properties—such as electronic band structure, density of states, charge density distribution, real and imaginary parts of the dielectric functions, joint density of states (JDOS) and the optical absorption and reflection rates according to the energy of incident light—were investigated and evaluated. The electronic band structure was computed using both the generalized gradient approximation Perdew–Burke–Ernzerhof and HSE06 methods. The results show that the ASiGeNR configurations have a direct bandgap, ranging from a minimum of 0.0889 to a maximum of 0.7528 eV, depending on the number of atoms along the nanoribbon width, with the bandgap opening at the Γ point. There is a hybridization of sp² and sp³ orbitals in the ASiGeNRs, with the σ bonds being relatively stronger than the π bonds. ASiGeNR systems allow electromagnetic waves to pass through, primarily in the *z*-direction. The energy levels and directions in which the ASiGeNR structure absorbs or disperses the most energy occur between 2 and 4 eV in the *y*- and *z*-directions. The real part of the dielectric function varies significantly across the different structures, with the most noticeable change occurring in the *z*-direction. The ASiGeNR6 structure exhibits the highest absorption peak, which gradually decreases in the subsequent structures. The imaginary part of the dielectric function tends to peak at photon energies below 4 eV, indicating strong interaction between light and the material structures within this energy range. This suggests that changes in nanoribbon width significantly affect the optical properties of the material. The JDOS results align with the optical absorption spectrum, with peaks in JDOS corresponding to peaks in the optical absorption spectrum.

## 1. Introduction

Nanoribbon materials based on C, Si, P and Ge are highly intriguing due to their wide applicability across various fields. The first nanoribbon material to attract interest was graphene nanoribbons. Since 2009, Faccio *et al*. [1] have studied the mechanical properties of graphene nanoribbons. That article presents the structural, electronic and mechanical properties of zigzag graphene nanoribbon materials using density functional theory (DFT) calculations. The results show that the mechanical properties of the material change linearly within a certain limited range of uniaxial stresses in the cyclic direction, with nonlinear changes occurring at higher distortions. That study also confirms the potential application of graphene nanoribbons in nanoelectronic devices. Subsequently, Celis *et al*. [2] conducted studies related to the properties, fabrication and applications of graphene nanoribbons. That study uses both top-down and bottom-up approaches to fabricating graphene nanoribbons using lithography, catalytic cutting, chemical methods and epitaxy, comparing their electronic properties. Alongside graphene nanoribbons, research on silicene nanoribbons has also flourished. In 2008, Sun *et al*. [3] studied the electronic and structural properties of SiC nanoribbons. The results showed that armchair SiC nanoribbons are non-magnetic semiconductors, whereas zigzag SiC nanoribbons are magnetic metals. The electronic properties of the SiC nanoribbons were studied in detail using first-principles calculations and the generalized gradient approximation (GGA) method [4]. Zigzag SiC nanoribbon configurations with widths of 2−4 atoms exhibit metallicity with a small bandgap. For SiC armchair nanoribbons, the p-orbitals of C and Si primarily contribute near the Fermi level, and the bandwidth of the SiC armchair is larger than that of the zigzag SiC nanoribbons [5,6]. The electronic and optical properties of SiC, penta-SiC2 and g-SiC3 nanoribbons have been studied in detail in several other works [7,8]. The structural, electronic and magnetic properties of silicene nanoribbons adsorbing alkali metal atoms have also been thoroughly examined [9,10].

More recently, nanoribbon structural systems based on the combination of Si and Ge, known as siligenes, have garnered significant interest due to their wide application potential in the fields of sensors and nanoelectronics [11–13]. Initial results show that bidirectional HSiGe is a semiconductor with a bandgap of approximately 0.6 eV. The findings also indicate that two-dimensional SiGe remains dynamically stable under tensile distortions of 4–6%. Unlike other heavy-metal-based two-dimensional systems such as silicene and phosphorene [14–16], SiGe has a sufficiently large bandgap, making it suitable for spintronics and applications based on the quantum spin Hall effect. The thermodynamic state of two-dimensional SiGe reveals medium/low energy barriers for alkali metal atoms, ranging from 0.14 to 0.35 eV, making it promising for use in fast-charging devices and stable voltage storage.

The electronic and magnetic properties of monolayer SiGe have been investigated and improved through oxidation and hydrogenation processes [17]. The results indicate that oxidation does not alter the magnetic properties of monolayer SiGe but introduces a bandgap of up to 1.35 eV, depending on the oxygen concentration. In contrast, hydrogenation induces ferromagnetic properties, primarily contributed by Ge atoms, and significantly widens the bandgap, making the monolayer SiGe a magnetic semiconductor. These findings suggest that chemical functionalization with oxygen and hydrogen can render monolayer SiGe a potential two-dimensional material for optoelectronic and spintronic applications. The electronic, optical and thermal properties of germanene monolayers under the influence of an external electric field were also studied [18]. The results show that the bandgap opens under an electric field and increases linearly with field intensity. Thermal conductivity and heat capacity increase with temperature due to the higher thermal energy of charge carriers. The thermal properties of germanene under zero bias differ significantly from those under an applied electric field. The light absorption and electronic properties of the black and green phases of monolayer SiSe under biaxial strain were also examined [19]. The results reveal that the black phase of SiSe has an indirect bandgap of 1.11 eV (Perdew–Burke–Ernzerhof (PBE)) and 2.94 eV (HSE06). The bandgap increases under tensile strain and decreases under compressive strain. For the green phase, the indirect bandgap is 0.62 eV (PBE) and 2.12 eV (HSE06), with the bandgap decreasing under both compressive and tensile strain. The black phase transitions from a semiconductor to a metal under approximately −4% compressive strain, while the green phase retains its semiconducting nature under strains ranging from −6% to 6%. These results demonstrate the tunability of the electronic and optical properties of monolayer SiSe through biaxial strain. The structural, electronic and optical properties of monolayer GeS and SiS under biaxial strain were also explored [20]. Both GeS and SiS monolayers exhibit indirect bandgaps. The bandgap of GeS reaches a maximum under −2% compressive strain and decreases with further compression or stretching. In terms of optical properties, both monolayers show strong light absorption in the ultraviolet region and partially in the visible light spectrum. With increased tensile strain, absorption in the visible light region is enhanced and extended due to a redshift in the absorption edge. These findings suggest that biaxial strain can effectively tune the electronic and optical properties of GeS and SiS monolayers, making them promising candidates for flexible electronics and optical applications. The phonon and electronic transport properties of halogenated SiGe monolayers were studied in detail [21]. The results confirm the full dynamic stability of SiGe and F2-SiGe monolayers, while SiGe monolayers halogenated with Cl and Br were dynamically unstable. Halogenation transforms the semi-metallic nature of SiGe monolayers into semiconducting properties. The Seebeck coefficient increases, and the lattice thermal conductivity decreases after halogenation, leading to an improved thermoelectric figure of merit (ZT). The ZT at room temperature for SiGe monolayers increases from 0.112 to 0.737 after fluorine atoms are added. The study highlights that halogenation of two-dimensional materials can enhance their thermoelectric properties.

Besides SiGe two-dimensional materials, the application potential of the one-dimensional SiGe structure is also significant, though it has not yet been fully and comprehensively studied. The one-dimensional SiGe materials are also promising materials for optoelectronic applications, thermoelectric devices or as components in next-generation transistors.

In this study, the electronic, magnetic and optical properties of the armchair SiGe nanoribbon (ASiGeNR) configuration were investigated to assess the applicability of these materials.

## 2. Material and methods

**Figure 1.**
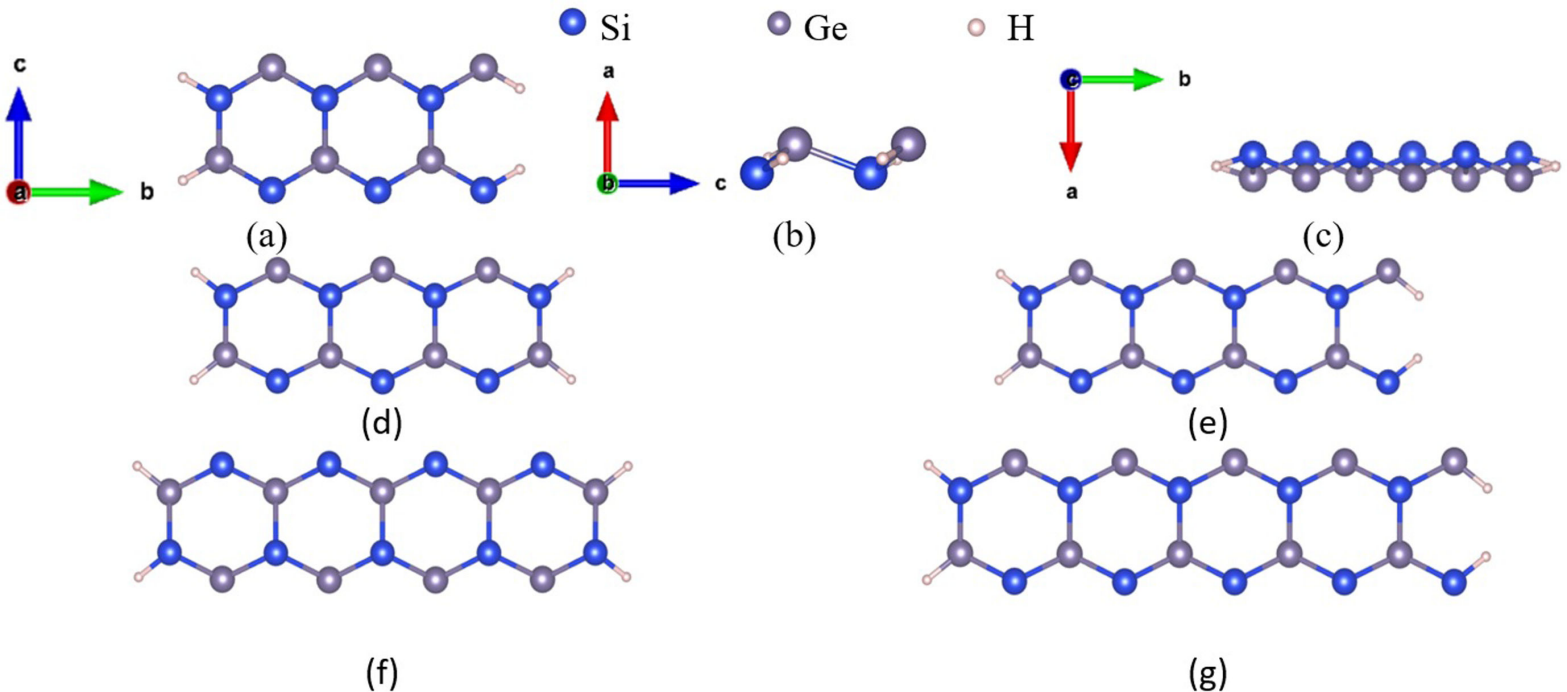
The optimal structure of ASiGeNR6, 7, 8, 9, 10. (*a*) ASiGeNR6 in the direction a, from the top down; (*b*) ASiGeNR6 in direction b, from the side edge; (*c*) ASiGeNR6 in the direction c, from the side edge. (*d*) ASiGeNR7 in the direction a, from the top down; (*e*) ASiGeNR8 in the direction a, from the top down; (*f*) ASiGeNR9 in the direction a, from the top down; (*g*) ASiGeNR10 in the direction a, from the top down.

The calculations were performed on five configurations, with ribbon widths of 6, 7, 8, 9 and 10 atoms, denoted as ASiGeNR6, ASiGeNR7, ASiGeNR8, ASiGeNR9 and ASiGeNR10, respectively. The unit cell of ASiGeNR6 was composed of six Si atoms, six Ge atoms and four functional H atoms at the side edges to enhance the stability of the structure. Similarly, the other structures had an increasing number of atoms corresponding to their width. To study the electronic and optical properties of the structures, DFT calculations were employed using the Vienna Ab initio Simulation Package (VASP) [22,23]. The exchange-correlation energies of electrons were computed based on the GGA [24], utilizing the PBE functional and the projector-augmented wave method [25]. To further clarify the PBE calculation results, the electronic band structure was also computed using the Heyd–Scuseria–Ernzerhof (HSE06) hybrid functional [26]. The wave functions and energy states were constructed from a plane-wave basis set with a maximum energy cutoff of 500 eV. The k-point grids in the Monkhorst–Pack scheme used for structural optimization calculations were 1 × 1 × 8. The system was optimized until the forces were less than EDIFFG = −10^−8^ eV Å^−1^, as per the Hellmann–Feynman force limit. The energy convergence was set to EDIFF = 10^–8^ eV between consecutive ionic steps. The optimized unit cell structure of the system is shown in figure 1. Self-consistent calculations were performed after structural optimization, with the k-points grid extended to 1 × 1 × 100. The electronic band structure, density of states (DOS), charge density distribution, spin density distribution and optical properties were subsequently investigated.

After the optimization process, the basic structural parameters were also determined. In the case of ASiGeNR6, the bond length between the nearest Si and Ge atoms is 2.32744 Å, between the nearest Si atoms is 3.69313 Å, and between the nearest Ge atoms is 3.69539 Å; the bond angles are approximately 106.76240° and 106.51880°, corresponding to the Si and Ge vertices, respectively; the buckling of the structure is approximately 0.79966 Å. The ASiGeNR7, ASiGeNR8, ASiGeNR9 and ASiGeNR10 structures have parameters that are very similar to those of ASiGeNR6. To evaluate the structural stability, the formation energy was determined. The formation energy (denoted as *E*_for_) is calculated as the total energy of the SiGe structure (*E*_SiGe_) minus the free energies of all constituent atoms, as in equation (2.1):


(2.1) $$E_{for}=E_{SiGe}-n_{Si}E_{Si}-n_{Ge}E_{Ge}.$$

Here, *n*_Si_ and *n*_Ge_ are the atomic numbers of Si and Ge in the structure, and *E*_Si_ and *E*_Ge_ are the free energies of Si and Ge. The results show that *E*_for_ is approximately −12.89 eV with all configurations. This indicates that the ASiGeNR structure is thermodynamically very stable, easy to form and highly likely to exist.

The optical properties of the structure are investigated through the dielectric function. The dielectric function consists of two parts: the real part and the imaginary part, which are expressed through equation (2.2):


(2.2) $$\varepsilon (\omega )=\varepsilon_{1}(\omega )+i\varepsilon_{2}(\omega ).$$

Here, *ε*_1_(*ω*) represents the real part and *ε*_2_(*ω*) represents the imaginary part. Many other optical properties, such as the absorption coefficient and the reflectivity coefficient, are derived from the dielectric function. In VASP, the dielectric function is read from the vasprun.xml file, which allows the calculation of optical properties at the GW0 + BSE level.

## 3. Results and discussion

### 3.1. The electronic band structures

**Figure 2.**
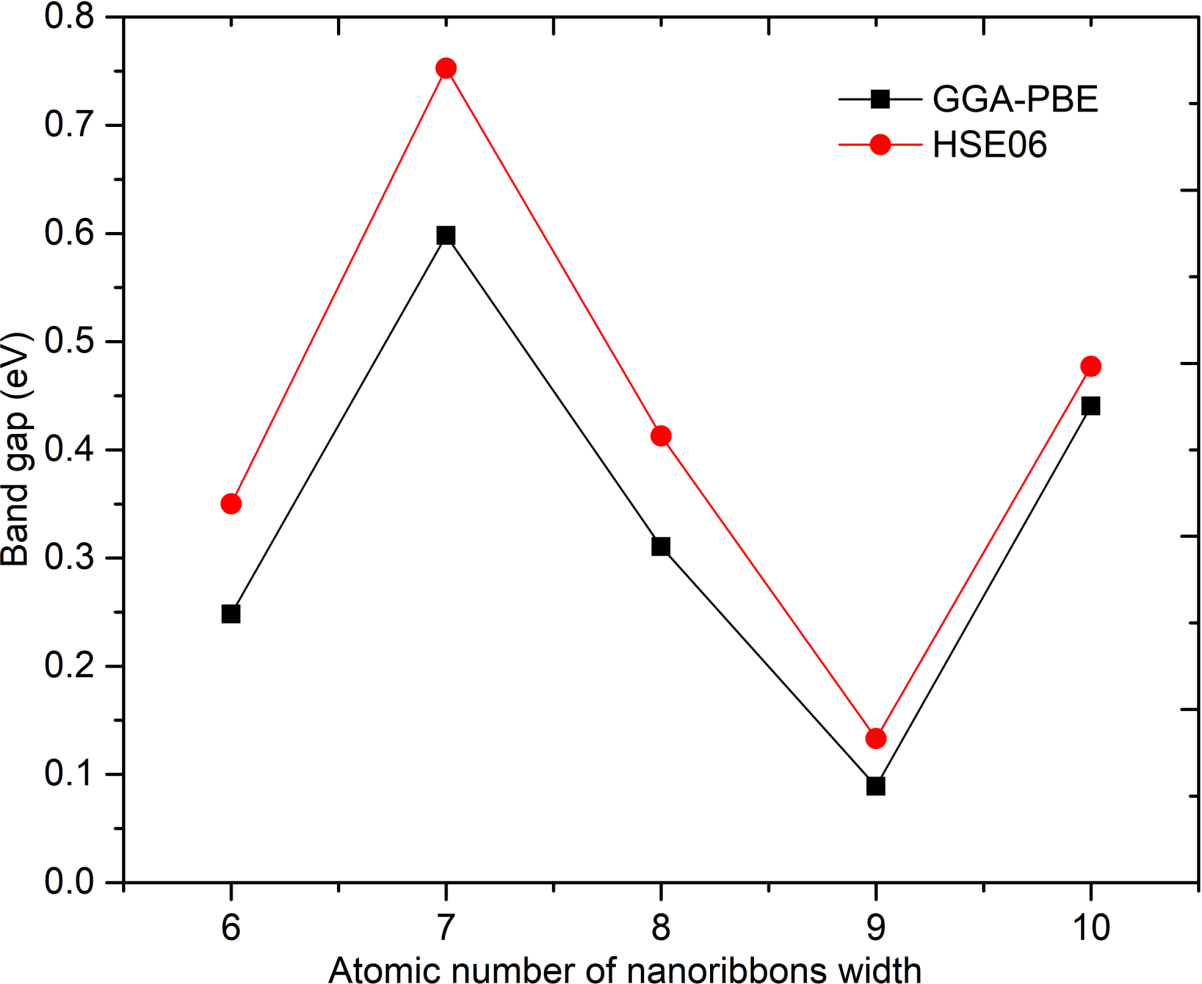
The bandgap of the structures was calculated using GGA-PBE and HSE06.

The results of the electronic band structure calculations show the existence of a bandgap in all structures, with relatively large differences depending on whether the GGA-PBE or HSE06 method was used. The specific results are shown in table 1 and figure 2.

**Table 1.** The bandgap values of the structures were calculated using both GGA-PBE and HSE06 methods.

	bandgap, *d* (eV)				
configuration	ASiGeNR6	ASiGeNR7	ASiGeNR8	ASiGeNR9	ASiGeNR10
GGA-PBE	0.2480	0.5980	0.3104	0.0889	0.4405
HSE06	0.3501	0.7528	0.4129	0.1331	0.4772
Δ*d*	0.1021	0.1548	0.1025	0.0442	0.0367

The results indicate that the bandgap reaches its maximum value for the ASiGeNR7 configuration, with values of 0.5980 eV using the GGA-PBE calculation and 0.7528 eV with HSE06. The bandgap reaches its minimum value for the ASiGeNR9 configuration, showing values of 0.0889 and 0.1331 eV, respectively, for the two methods. For configurations with an even number of atoms along the ribbon width, the bandgap tends to increase as the number of atoms increases. However, in configurations with an odd number of atoms, the bandgap seems to decrease as the number of atoms increases. This trend needs to be confirmed through further calculations on additional structures. The results also show that calculations using the HSE06 hybrid functional method consistently yield larger bandgaps compared to GGA-PBE. The difference between the bandgaps calculated by HSE06 and GGA-PBE is quite noticeable, especially for structures with larger bandgaps, such as ASiGeNR7. Meanwhile, for structures with smaller bandgaps, like ASiGeNR9, the difference between the two methods tends to be smaller. HSE06 is generally considered more accurate in predicting bandgaps as it accounts for electronic interactions more effectively, but it requires greater computational resources compared to GGA-PBE.

**Figure 3.**
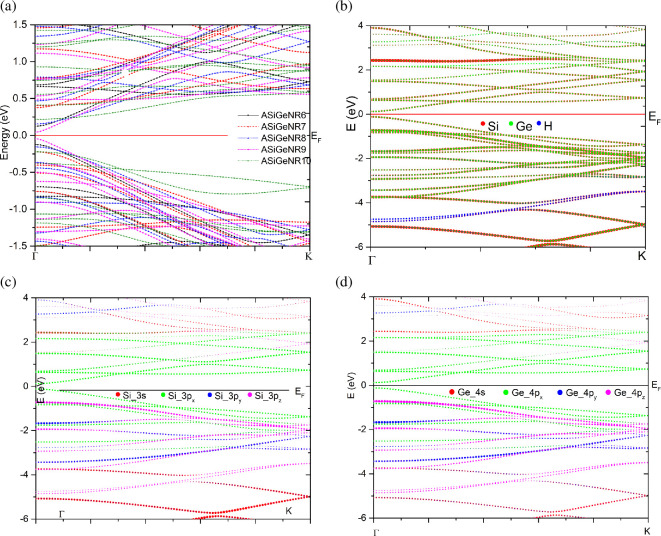
Electronic band structure of ASiGeNRs. (*a*) Total contribution of atoms of all configurations. (*b*) The contribution of each atom Si, Ge, H of ASiGeNR6. (*c*) The contribution of the 3s, 3p_*x*_, 3p_*y*_ and 3p_*z*_ orbitals of the Si atom of ASiGeNR6. (*d*) The contribution of the 4s, 4p_*x*_, 4p_*y*_ and 4p_*z*_ orbitals of the Ge atom of ASiGeNR6.

The electronic band structures of ASiGeNR are presented in figure 3. Figure 3*a* shows the total atomic contributions across all configurations, while figure 3*b* shows the contributions of individual Si, Ge and H atoms. Figure 3*c* presents the contributions from the 3s, 3p_*x*_, 3p_*y*_ and 3p_*z*_ orbitals of the Si atoms, and figure 3*d* displays the contributions from the 4s, 4p_*x*_, 4p_*y*_ and 4p_*z*_ orbitals of the Ge atoms of ASiGeNR6.

The results show that the ASiGeNR structures have a direct bandgap ranging from 0.09 to 0.61 eV, which opens at the Γ point. This indicates that ASiGeNRs exhibit semiconducting properties. The bandgap of ASiGeNRs, compared to armchair silicene nanoribbons (ASiNRs) and armchair germanene nanoribbons (AGeNRs), shows a relatively large difference, with values exceeding 0.4 eV [27]. Around the Fermi level, the contributions of Si and Ge are predominant, with the contribution of the Ge atoms being more pronounced than that of Si. The contribution of H is negligible in the electronic band structure and is primarily concentrated in the deep region below the valence band.

The orbital contribution analysis results show that the s, p_*x*_, p_*y*_ and p_*z*_ orbitals of both Si and Ge contribute similarly to the electronic band structure. The Si 3s and Ge 4s orbitals primarily contribute deep in the valence region, with minimal contribution in the conduction region. Around the Fermi level, the p_*x*_, p_*y*_ and p_*z*_ orbitals of both Si and Ge atoms are the main contributors. The Si 3p_*x*_ and Ge 4p_*x*_ orbitals contribute the most to the energy regions around the Fermi level, concentrated in energy levels from −1 to 2 eV, while the Si 3p_*y*_, Si 3p_*z*_, Ge 4p_*y*_ and Ge 4p_*z*_ orbitals primarily contribute to the region below the Fermi level, with energy levels ranging from −2.5 to −1.0 eV. In some regions, there is an overlap between the *s* and p_*x*_–p_*y*_ orbital contributions of both Si and Ge, which could result in a mixture of sp² and sp³ hybridization in the ASiGeNR structure. This was more clearly assessed based on the distribution of the electronic DOS.

### 3.2. The electronic density of states

**Figure 4.**
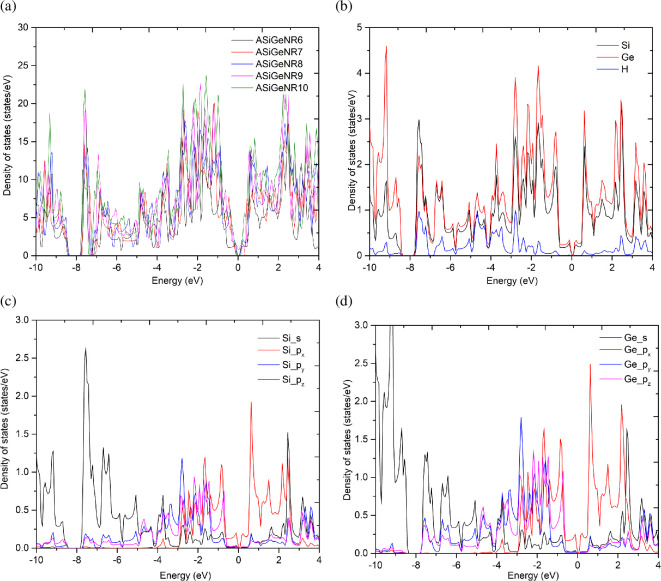
The DOS of ASiGeNR. (*a*) The total DOS of atoms of all configurations. (*b*) The DOS of Si, Ge and H of ASiGeNR6. (*c*) The DOS of the 3s, 3p_*x*_, 3p_*y*_ and 3p_*z*_ orbitals of the Si atoms of ASiGeNR6. (*d*) The DOS of the 4s, 4p_*x*_, 4p_*y*_ and 4p_*z*_ orbitals of the Ge atoms of ASiGeNR6.

Studying the electronic DOS provides a clearer assessment of the electronic properties derived from the band structure. The DOS is presented in figure 4.

Figure 4*a* shows the total DOS for atoms in all configurations; figure 4*b* presents the DOS for each Si, Ge and H atom; figure 4*c* illustrates the DOS for the 3s, 3p_*x*_, 3p_*y*_ and 3p_*z*_ orbitals of the Si atom; and figure 4*d* shows the DOS for the 4s, 4p_*x*_, 4p_*y*_ and 4p_*z*_ orbitals of the Ge atom in ASiGeNR6. The results show that the DOS is zero around the Fermi level, reaffirming the presence of a bandgap at this level. The DOS is concentrated mainly around the energy levels of −2 and +2 eV, with contributions primarily from Si and Ge, where Ge’s contribution is higher than Si’s at most energy levels, as shown in figure 4*b*. Figure 4*c,d* also shows that the contributions of the Si 3s and Ge 4s orbitals are mostly in the valence region, around −8 eV, while the p_*x*_, p_*y*_ and p_*z*_ orbitals dominate near the Fermi level. Additionally, a peak convolution between the s and p_*x*_–p_*y*_ orbitals around −2 eV further supports the assumption of mixed sp^2^ and sp^3^ hybridization in the ASiGeNR structure.

### 3.3. The charge density distributions

**Figure 5.**
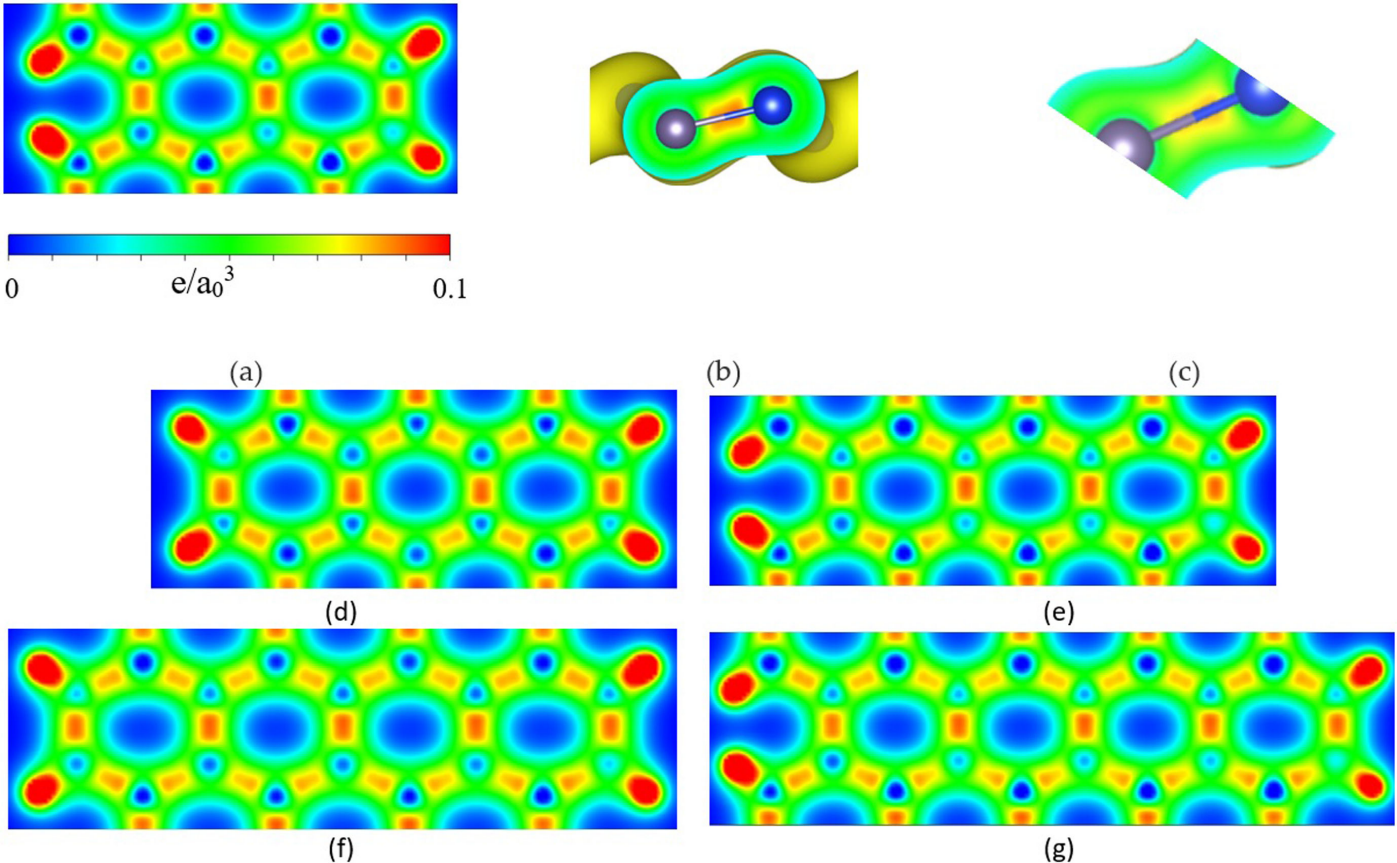
Charge density distributions of (*a*) ASiGeNR6 structure and (*b*) ASiGeNR6 structure between two Si and Ge atoms on the proximal edge of the hexagonal ring; (*c*) ASiGeNR6 structure, between two Si and Ge atoms on the far edge of the hexagonal ring; (*d*) ASiGeNR7 structure; (*e*) ASiGeNR8 structure; (*f*) ASiGeNR9 structure; (*g*) ASiGeNR10 structure.

The charge density distribution provides an assessment of the strength of the chemical bonds in the structure. Figure 5 shows the charge density distributions in various sections. Figure 5*a* shows the charge density distribution of the ASiGeNR6 structure, figure 5*b* presents the charge distribution between two Si and Ge atoms at the proximal edge and figure 5*c* displays the charge distribution between two Si and Ge atoms at the distal edge of the hexagonal ring in the ASiGeNR6 structure. Figure 5*d−g* depicts the charge density distributions of the ASiGeNR7, ASiGeNR8, ASiGeNR9 and ASiGeNR10 structures, respectively. The results show that the σ bonds involving Si 3s, Si 3p_*x*_, Si 3p_*y*_, Ge 4s, Ge 4p_*x*_ and Ge 4p_*y*_ orbitals are relatively stronger than the π bonds formed by the Si 3p_*z*_ and Ge 4p_*z*_ orbitals. Additionally, the σ bond between the two Si and Ge atoms at the proximal edge is more pronounced than that between the two Si and Ge atoms at the distal edge. The charge density distribution between different structures shows no significant variations, with the bond strength between the inner Si and Ge atoms being stronger than that between the outer Si and Ge atoms.

### 3.4. Optical properties

**Figure 6.**
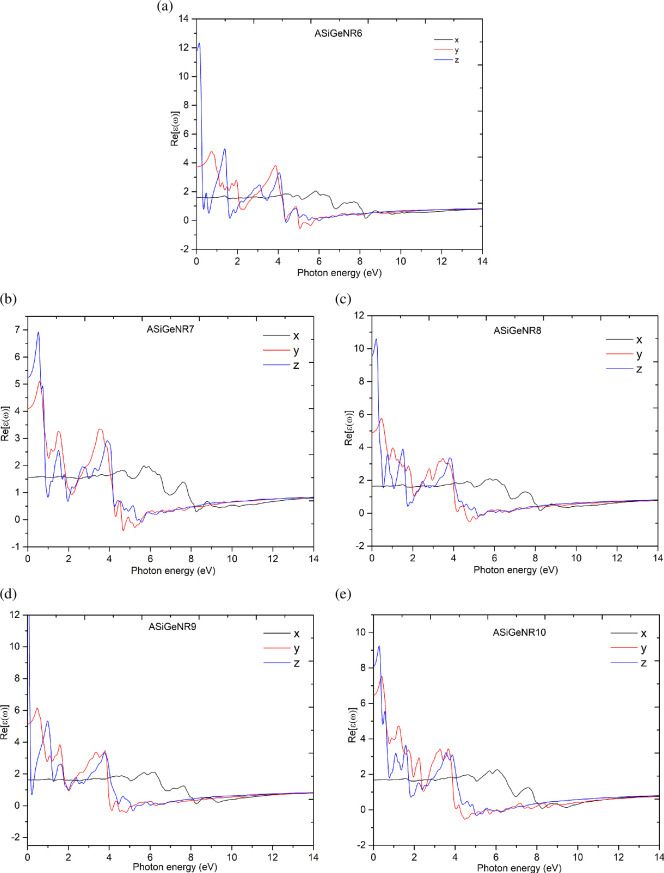
The real part of the dielectric function of (*a*) ASiGeNR6; (*b*) ASiGeNR7; (*c*) ASiGeNR8; (*d*) ASiGeNR9; and (*e*) SiGeNR10.

**Figure 7.**
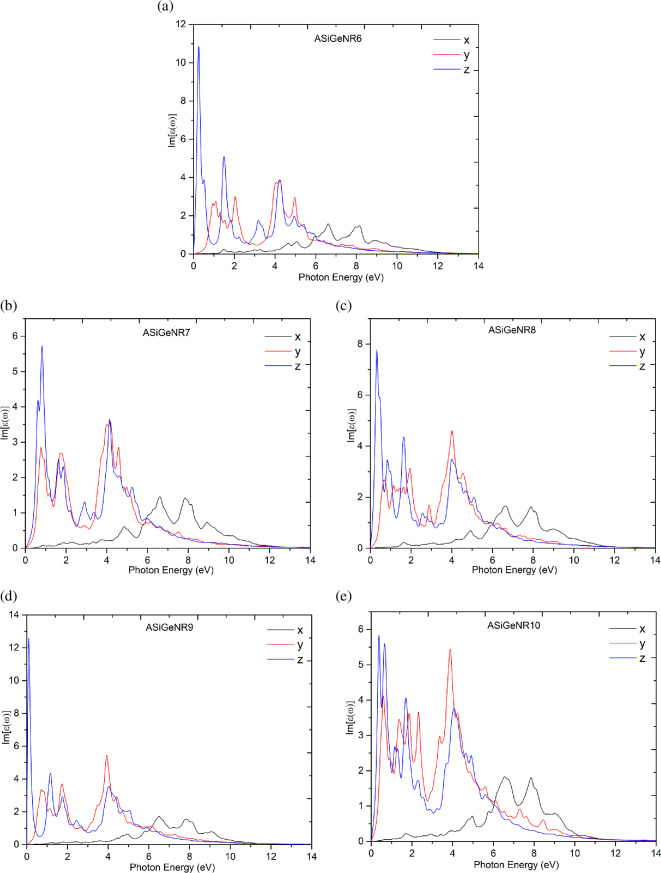
The imaginary part of the dielectric function of (*a*) ASiGeNR6; (*b*) ASiGeNR7; (*c*) ASiGeNR8; (*d*) ASiGeNR9; and (*e*) SiGeNR10.

**Figure 8.**
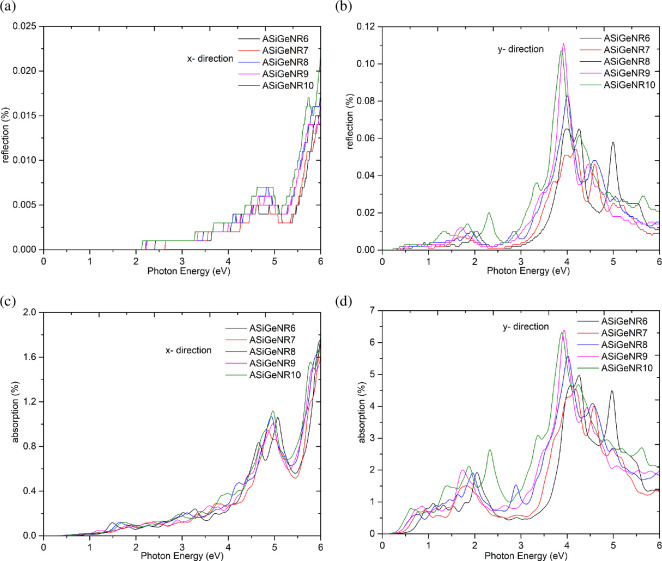
Optical reflection and absorption rate. (*a*) Optical reflection rate according to incident photon energy*—x*-direction. (*b*) Optical reflection rate according to incident photon energy*—y* direction. (*c*) Optical absorption rate according to incident photon energy*—x* direction. (*d*) Optical absorption rate according to incident photon energy—*y*-direction.

**Figure 9.**
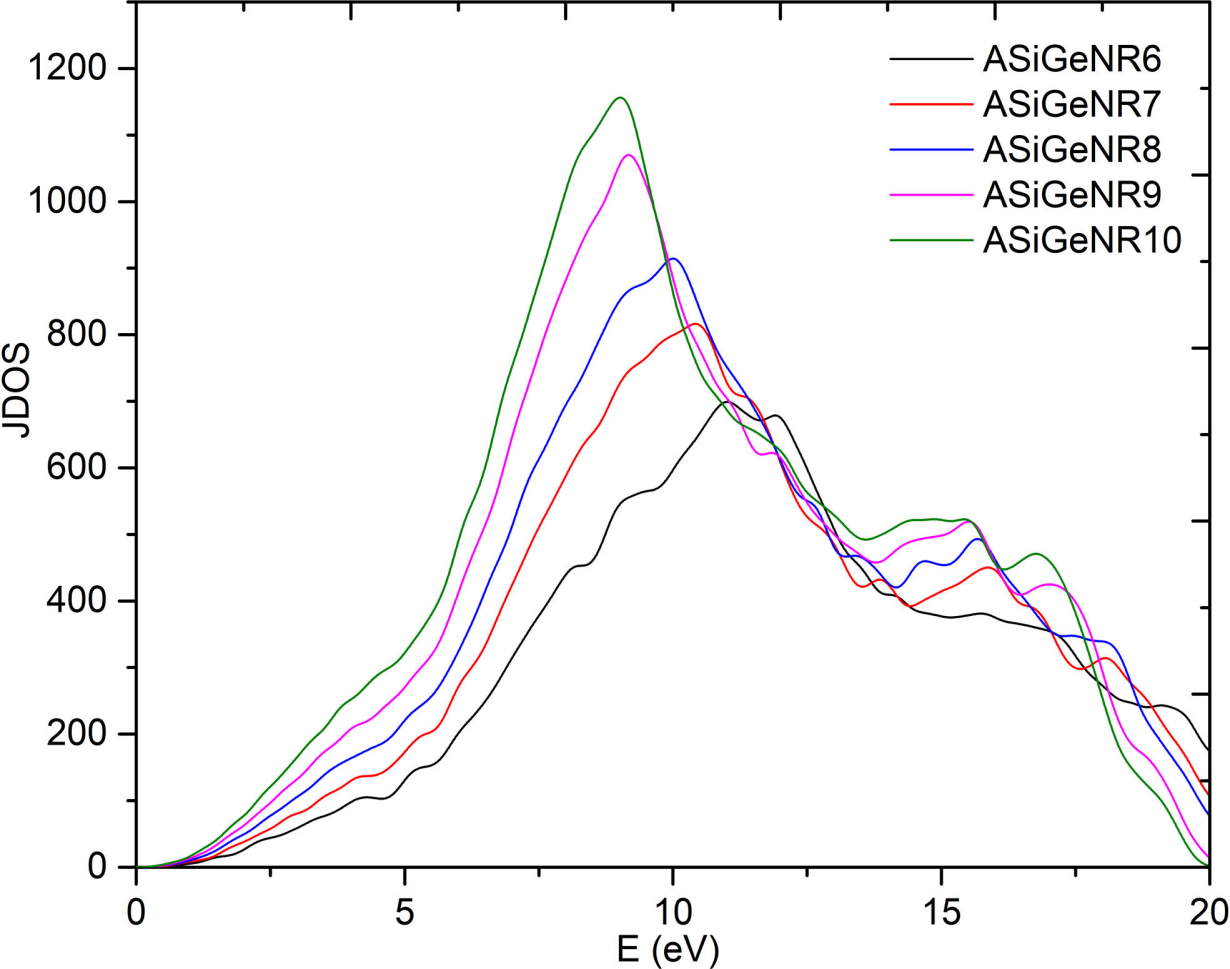
Joint density of states (JDOS) follows the incident photon energy.

Studying the optical properties of a structural system is crucial for accurately guiding its application in the field of optoelectronics. In this section, key characteristics affecting the optical properties of ASiGeNR are calculated and presented in figures 6–9.

Figure 6*a–e* shows the real parts of the dielectric function for ASiGeNR6, ASiGeNR7, ASiGeNR8, ASiGeNR9 and ASiGeNR10 in the *x*-, *y*- and *z*-directions, respectively. Figure 7*a–e* presents the imaginary parts for these configurations. Figure 8*a–d* shows the reflection and optical absorption ratios based on the incident photon energy. Figure 9 displays the results of calculating the joint DOS (JDOS) as a function of incident photon energy. The real part of the dielectric function helps evaluate the material’s polarization under the influence of an electromagnetic field, indicating its ability to store electric field energy, which in turn affects its optical properties. The results show that, in the low-energy photon region below 2 eV, large peaks appear along the *y*- and *z*-directions for all ASiGeNR structures, indicating strong light absorption in this low-energy range along these axes. In contrast, in the *x*-direction, no significant changes occur within this energy range, suggesting that the structures neither absorb light strongly nor react notably to low-energy photons along this axis.

In the energy range from 2 to 4 eV, the real part of the dielectric function tends to decrease and oscillates between positive and negative values, indicating that the material systems begin to exhibit light-reflecting properties, most notably along the *z*-direction. In the range from 4 to 6 eV, the curve continues to oscillate, suggesting alternating absorption and reflection of light depending on the frequency. The curves along the *y*- and *z*-directions remain relatively small in this range, while the *x*-curve remains relatively stable at energy levels below 6 eV. In the range from 6 to 10 eV, the real part of the dielectric function approaches zero in all three directions, indicating that at higher energy levels, the material becomes less capable of absorbing or reflecting light, essentially becoming more transparent. Beyond 10 eV, the curves stabilize, with values nearly reaching zero, suggesting that the material does not strongly interact with light in the high-energy region. Overall, the real part of the dielectric function shows significant changes across different structures, particularly in the *z*-direction; the ASiGeNR6 structure exhibits the highest absorption peak, which gradually decreases in subsequent structures. This indicates that variations in the nanoribbon width relatively affect the material’s optical properties. The results also show a small negative region in the real part of the dielectric function, suggesting that the ASiGeNR systems can allow electromagnetic waves to pass through, primarily along the *y*- and *z*-directions.

Analysing the imaginary part of the dielectric function further clarifies the interaction between the material systems and light. For ASiGeNR6, the imaginary part shows peaks around 1.5 and 4.2 eV in the *y-* and *z*-directions and peaks at 6.6 and 8.0 eV in the *x*-direction. These represent the energy levels and directions where the ASiGeNR structure absorbs or disperses the most energy. For nearly all structures, the imaginary part tends to peak at photon energies below 4 eV, indicating strong interactions between light and the material structures in this energy range. In most cases, the *z*-direction exhibits higher light absorption compared to the *x*- and *y*-directions. This suggests that the material is anisotropic, with its interaction with light varying depending on the photon direction. However, in some structures, such as ASiGeNR8, the imaginary part in the *y*-direction shows a stronger peak than in the *z*-direction, highlighting variability in optical properties due to changes in the structure and the nanoribbon width.

The results of the optical reflection spectrum study show that all structures exhibit increased reflection at photon energies of 4 eV and above. Specifically, the ASiGeNR9 and ASiGeNR10 structures display higher reflection at photon energies greater than 4 eV but tend to have lower reflection compared with ASiGeNR6 at energies below 4 eV in the *x*-direction. In the *y*-direction, the structures reflect strongly at photon energies ranging from 1 to 4 eV, with distinct peaks; ASiGeNR10 exhibits the highest reflection in this range, while ASiGeNR6 and ASiGeNR7 show lower reflection. Notably, ASiGeNR9 and ASiGeNR8 also display clear differences in reflection peaks, indicating variations in the electronic structures of these materials. The optical absorption spectrum indicates that all structures tend to absorb light strongly in the range of 2–5 eV, with ASiGeNR8 and ASiGeNR9 demonstrating the highest absorption. However, ASiGeNR10 tends to absorb less than the other structures. The differences in the width and height of the absorption peaks may be related to variations in the size and structure of the nanoribbons in the *x*-direction. In the *y*-direction, the optical absorption spectrum shows higher absorption compared to the *x*-direction, particularly in the 2−4 eV range. ASiGeNR6 exhibits lower absorption compared to the other structures, while ASiGeNR8 and ASiGeNR10 show higher absorption. Thus, all five structures tend to reflect and absorb strongly in the photon energy range of 2–5 eV, suggesting potential applications in optoelectronic or photonic devices operating in this energy range. The distinct differences in reflection and absorption intensity between the structures indicate that small variations in nanoribbon structure (from ASiGeNR6 to ASiGeNR10) significantly affect their optical properties, especially in the low-energy range (below 4 eV). This can be linked to differences in electronic structure, energy band characteristics and the DOS.

JDOS is an important function in solid-state physics and material calculations, particularly in the study of optical and electronic properties. It represents the number of electronic states available for photon absorption or emission processes, based on transitions from the valence band to the conduction band. JDOS provides critical insight into how materials interact with light, making it a vital tool for understanding and designing optoelectronic devices.

The JDOS graphs illustrate that the production of electron–hole pairs peaks at photon energies around 10−15 eV, indicating a higher likelihood of electron transitions between bands at these energy levels. Among the structures, ASiGeNR6 exhibits the lowest JDOS across most energy ranges, suggesting a reduced availability of electronic states for transitions compared to its wider counterparts. As the width of the nanoribbons increases (e.g. ASiGeNR7 and ASiGeNR8), the JDOS also rises, with ASiGeNR10 displaying the highest JDOS across most energy levels. This implies that this particular nanoribbon has a greater propensity for electron transition processes. Notably, the results from JDOS correlate well with the optical absorption spectra, where peaks in JDOS align with peaks in the optical absorption spectrum. This relationship highlights the materials’ capacity to absorb photons at specific energy levels, thereby elucidating their interaction with light.

## 4. Conclusion

Using DFT, ASiGeNR structures with varying ribbon widths were investigated through the VASP computational simulation program. The study demonstrated key electrical and optical properties of the system, including the electronic band structure, DOS, charge density distribution and both the real and imaginary parts of the dielectric functions, as well as JDOS spectroscopy, optical absorption and reflection in relation to incident photon energy. The presence of a direct bandgap, which varies with ribbon width for each structure and opens at the Γ point, suggests significant potential for applications in nanoelectronics. Specific photon energy regions for absorption and reflection were identified, which could guide the development of optoelectronic applications. These findings provide a crucial foundation for the application orientation of ASiGeNR material systems in nanoelectronics, optoelectronics, thermoelectric devices or as a component in next-generation transistors. However, further detailed studies are necessary to validate these results.

## Data Availability

All data needed to evaluate the conclusions in the paper are available in the Dryad Digital Repository [28].
